# A Possible Missing Link Between Obstructive Sleep Apnea Syndrome (OSA) Associated with Tobacco Use and Inflammation Biomarkers

**DOI:** 10.3390/healthcare13101177

**Published:** 2025-05-19

**Authors:** Adriana-Loredana Pintilie, Andreea Zabara Antal, Bogdan-Mihnea Ciuntu, David Toma, Raluca Tiron, Ruxandra Stirbu, Mihai Lucian Zabara, Radu Crisan Dabija

**Affiliations:** 1 Clinical Hospital of Pulmonary Diseases Iași, 700116 Iasi, Romania; pintilie.adriana.loredana.mg6.7@gmail.com (A.-L.P.); tomadavidro@gmail.com (D.T.); raluca-teodora.tiron@email.umfiasi.ro (R.T.); stirbu.ruxandra@email.umfiasi.ro (R.S.); radu.dabija@umfiasi.ro (R.C.D.); 2 Faculty of General Medicine, Grigore T. Popa University of Medicine and Pharmacy Iasi, 700115 Iasi, Romania; 3 Surgery Department, Faculty of General Medicine, Grigore T. Popa University of Medicine and Pharmacy Iași, 16 Universității, 700115 Iasi, Romania; bogdan-mihnea.ciuntu@umfiasi.ro (B.-M.C.); mihai-lucian.zabara@umfiasi.ro (M.L.Z.); 4 Department of General Surgery and Liver Transplantation, St. Spiridon University Hospital Iași, 700115 Iasi, Romania; 5 Pulmonary Department, Faculty of General Medicine, Grigore T. Popa University of Medicine and Pharmacy Iași, 700115 Iasi, Romania

**Keywords:** smoking, obstructive sleep apnea, inflammation, C-reactive protein (CRP), erythrocyte sedimentation rate (ERS)

## Abstract

**Background:** Obstructive sleep apnea (OSA) is increasingly recognized as a chronic condition associated with systemic low-grade inflammation. Elevated levels of inflammatory markers such as C-reactive protein (CRP), fibrinogen, TNF-α, and IL-6 have been observed in OSA patients, independent of obesity. Tobacco use, a known pro-inflammatory factor, may further exacerbate this burden. This study aimed to evaluate whether smoking influences inflammatory markers and OSA severity in newly diagnosed patients. **Methods:** We conducted a retrospective, observational study on individuals newly diagnosed with OSA between 1 January 2024 and 31 December 2024 at the Clinical Hospital of Pulmonary Diseases Iași. All participants underwent overnight respiratory polygraphy using the SleepDoc Porti 9 system (Löwenstein Medical), with OSA severity classified according to the American Academy of Sleep Medicine (AASM) criteria. Inflammatory status was assessed using CRP and the erythrocyte sedimentation rate (ESR). Smokers were defined as individuals who had smoked within the past year; non-smokers had a lifetime history of fewer than 50 cigarettes. Statistical analysis was performed using IBM SPSS Statistics. **Results:** Smokers (n = 55) shoation Index (ODI) values, compared to non-smokers (n = 49): AHI 45.29 ± 20.94 vs. 38.40 ± 19.84 events/hour, ODI 45.69 ± 21.05 vs. 38.44 ± 19.40 events/hour (*p* < 0.05 for both). Mean CRP levels were approximately 3.5 times higher in smokers (10.32 ± 11.69 mg/dL) than in non-smokers (2.97 ± 2.45 mg/dL), indicating a significantly elevated inflammatory burden. **Conclusions:** The inflammatory burden and clinical severity of OSA may be influenced by smoking. Routine inflammatory marker screening, particularly CRP, may improve risk stratification and treatment planning in OSA patients, especially those who smoke or are obese. Routine assessment of CRP and other inflammatory markers may improve risk stratification and guide personalized treatment strategies, particularly in smokers and obese patients with OSA.

## 1. Introduction

Still insufficiently debated and explored, sleep medicine has nonetheless been gaining increasing ground in recent years, compared to previous decades. The quality and quantity of sleep have become increasingly relevant topics. In the context of today’s society, marked by a drive for progress and a fast-paced lifestyle, sleep disorders are becoming more prevalent. Stress and poor dietary habits contribute to this obesity epidemic, which continues to rise unabated. Obesity stands as the primary risk factor for the development of sleep apnea syndrome, the most common condition in this field, associated with significant morbidity and mortality rates. In general, the daytime symptoms characteristic of sleep disorders gradually lead to the withdrawal of patients from their socio-professional environments and result in high medical costs. Even more so, due to the associated pathologies it generates and sustains, sleep apnea is the nocturnal disorder that most frequently turns the patient into a burden on society.

The prevalence of obstructive sleep apnea (OSA) in the general adult population has been reported to vary between 9% and 38%, with rates ranging from 13% to 33% in men and 6% to 19% in women [1]. These values tend to be significantly higher among older individuals [2]. Although the prevalence of OSA differs across countries and regions, estimates suggest that approximately 1 billion adults aged 30–69 years worldwide may be affected, with nearly 425 million experiencing moderate to severe forms [3]. Considering the expansion of the obesity epidemic in the general population, we anticipate an increase in the number of OSA patients. The condition is characterized by intermittent upper airway obstruction during sleep, leading to intermittent hypoxia and sleep fragmentation. The consequences extend beyond symptoms like snoring, headaches, daytime hypersomnolence, and poor sleep quality to cardiovascular, cerebrovascular, and metabolic diseases. When we talk about sleep apnea, we talk about multidisciplinary issues [4]. The repetitive episodes of hypoxia–reoxygenation characteristic of OSA negatively affect tissues throughout the body, leading to endothelial dysfunction and an amplified systemic inflammatory response [5]. The pathophysiology of OSA encompasses multiple harmful consequences, notably increased oxidative stress, sympathetic overactivation, endothelial dysfunction, hypercoagulability, metabolic disturbances, endocrine dysregulation, and chronic systemic inflammation [6]. Evidence increasingly supports a paradigm shift in which OSA should be considered a low-grade chronic systemic inflammatory disorder rather than merely a sleep-related breathing abnormality [7]. Erythrocyte sedimentation rate (ESR) and C-reactive protein (CRP) have mainly been used as screening tools for hidden inflammation and to assist in the diagnosis and follow-up of chronic diseases. The erythrocyte sedimentation rate (ESR), a non-specific marker of inflammation, is significantly higher in the severe OSA group than in the mild or moderate OSA group in most studies. However, there are no statistical differences in hs-CRP (high-sensitivity C-reactive protein) levels between the moderate and severe OSA groups. In contrast, in the mild OSA and control groups, the values are significantly lower in most articles addressing the subject [8]. Individuals with OSA or overlap syndrome (OS), defined as the coexistence of chronic obstructive pulmonary disease (COPD) and sleep apnea, exhibit higher levels of inflammatory markers, such as hs-CRP, interleukin-6 (IL-6), and granulocyte colony-stimulating factor (G-CSF), compared to those with OSA alone. Current literature shows that both OSA and overlap syndrome groups have higher inflammatory marker levels than healthy individuals in most studies evaluating this topic [9]. This inflammatory cascade is evidenced by elevated levels of specific biomarkers that indicate disease severity and potential therapeutic targets. Understanding the relationship between OSA and these inflammatory mediators provides crucial insights into the mechanisms underlying the increased cardiovascular and metabolic morbidity observed in affected patients. Studies have shown that OSA patients demonstrate higher levels of inflammatory factors (CRP, fibrinogen, TNF-α, IL-6), regardless of obesity status. The inflammatory cascade activation through adaptive mechanisms in OSA potentially represents a key molecular pathway underlying cardiovascular and metabolic disease development [10].

### 1.1. Inflammation in OSA—Still an Incompletely Elucidated Issue?

Tobacco use, a well-known risk factor for a range of respiratory and cardiovascular diseases, has been linked to the exacerbation of inflammation in various conditions, including chronic obstructive pulmonary disease (COPD) and cardiovascular disease. The effects of smoking on the respiratory system, such as airway remodeling and increased oxidative stress, are well documented. However, the interplay between tobacco use, OSA, and systemic inflammation remains insufficiently explored. Tobacco smoke has been shown to influence the immune system, possibly enhancing the inflammatory response induced by OSA. Combining these two factors may create a synergistic effect, potentially worsening the inflammatory burden and contributing to an accelerated progression of OSA-related complications. Beyond the widely known effects of smoking on lung function, it also impacts the function of the upper airways by inducing oropharyngeal narrowing and increasing the severity of OSA. Cigarette smoking was linked to variations in systemic inflammatory markers. An analysis of 78 immune, inflammatory, and metabolic markers linked current smoking status with increased values of several markers, including CRP [11]. When comparing the inflammatory levels of smokers and non-smokers diagnosed with OSA, studies found higher levels of inflammatory markers, such as CRP, haptoglobin, ceruloplasmin, and triglycerides, in smokers after a minimum of five years of monitoring [12]. Although several studies have attempted to establish a relationship between OSA and smoking, the existing data remain controversial. A study involving 3791 patients found that the pack-year number was higher in individuals diagnosed with severe OSA. Duration and smoking intensity correlated with the severity class of the sleep disorder. Among current smokers, the prevalence of moderate OSA was higher, whereas former smokers were more frequently found in the moderate and severe OSA groups. Additionally, the univariate analysis showed that current smokers were 1.2 times more likely to develop OSA, compared to new or former smokers. Former smokers had a 1.49-times higher likelihood of presenting OSA, compared to never smokers. In gender-stratified multivariate analysis, no significant associations were noticed. However, no independent effect of smoking on OSA was found. Instead, tobacco consumption appeared to be higher in individuals with more severe OSA, primarily when associated with cardiovascular comorbidities such as hypertension and coronary artery disease [6].

Obesity is not only the main factor in OSA development. There is a bidirectional relationship wherein OSA exacerbates weight gain. Particularly, visceral adiposity is a state of chronic inflammation due to the expansion of adipose tissue. Adipocytes and infiltrating macrophages release pro-inflammatory cytokines, chemokines, and adipokines, which amplify systemic inflammation. Intermittent hypoxia and sleep disruption both stimulate further cytokine production. Studies consistently demonstrate that obese individuals with OSA exhibit higher levels of inflammatory markers compared to non-obese counterparts or obese individuals without OSA, underscoring the synergistic effect of these conditions. Tumor necrosis factor-alpha, a pro-inflammatory cytokine, promotes insulin resistance and endothelial dysfunction, both prevalent in OSA [6]. Vgontzas et al. found elevated levels in obese patients with OSA, compared to obese controls without OSA. There was a perfect correlation between TNF-alpha factor levels and AHI or BMI [13]. Ciftci et al. identified higher IL-6 levels in obese OSA groups, compared with non-obese OSA patients or healthy controls [14]. Also, there was a link between the apnea severity class and the BMI. It was proved that continuous positive airway pressure (CPAP) therapy reduces IL-6 levels. Sleep fragmentation and hypoxia may induce leptin resistance, further driving inflammation. There are elevated leptin levels in obese OSA patients, with a decrease post-therapy, depending on the sleep disturbance severity [15]. Adiponectin, an anti-inflammatory adipokine, is reduced in obesity due to adipose tissue dysfunction. Its protective effects on insulin sensitivity and vascular health are dismissed in OSA [16].

Smoking generates chronic inflammation of the upper airway, contributing to OSA symptoms [17]. Kim et al. also found that patients with moderate to severe OSA present increased thickness and edema of the uvular mucosa in upper airway mucosal biopsies [18]. Additionally, Conway et al. observed that smokers exhibited a higher arousal index, longer respiratory events, and greater desaturation, thus contributing to the pathophysiological pathways of sleep apnea-related inflammation [19]. Inflammation of the upper airway mucosa can be evaluated by analyzing the cellular composition of induced and nasal sputa in non-smoking individuals. A study involving 24 non-smoking participants was conducted to evaluate the impact of CPAP usage. At baseline, individuals diagnosed with OSA presented a highly neutrophilic nasal inflammation profile. However, after 1, 10, and 60 days of therapy, the cellular profile of the induced sputum remained unchanged, indicating that appropriate CPAP therapy did not alter it [20].

This study aimed to investigate the potential link between tobacco use and inflammation biomarkers in patients with OSA by monitoring the levels of ESR and CRP in a cohort of OSA patients, both smokers and non-smokers. Furthermore, this study was designed to identify if smoking status impacts inflammation severity, to determine any differences, to quantify the inflammatory markers, and to determine whether these changes are statistically significant. Moreover, this study aimed to find out potential correlations between smoking status, body mass index, and gender with the decrease in CRP and ESR values following treatment. In the end, the paper aimed to assess the extent to which the results of the conducted study correlate with literature data.

### 1.2. Study Design, Participants, and Data Collection

This research was designed as a two-phase study to evaluate the inflammatory status of patients newly diagnosed with obstructive sleep apnea (OSA) and to assess the effects of continuous positive airway pressure (CPAP) therapy over time.

In the first phase, we retrospectively analyzed the medical records of newly diagnosed OSA patients admitted to the Clinical Hospital of Pulmonary Diseases Iași, between 1 January 2024 and 31 December 2024. We focused on assessing baseline inflammatory markers, specifically the C-reactive protein (CRP) and the erythrocyte sedimentation rate (ESR), along with sleep-related parameters obtained from polygraphy. Patients were further stratified by smoking status. The findings of this retrospective analysis are presented in this paper.

After evaluating the records, 104 patients met the eligibility criteria and were included in the study. These individuals were newly diagnosed with OSA and had complete data available for baseline inflammatory and clinical assessment.

Table 1 (left) outlines the inclusion criteria. A current smoker was defined as someone who had used cigarettes within the past year, whereas a never-smoker was defined as someone with a lifetime history of fewer than 50 cigarettes. Unfortunately, accurate data on pack-year consumption could not be consistently retrieved.

Out of a total of 460 patients, 356 were excluded due to one or more of the following: prior OSA treatment (e.g., at home), presence of acute infections (e.g., respiratory or urinary), unstable cardiovascular conditions, known chronic pulmonary diseases (e.g., COPD or asthma), autoimmune or inflammatory disorders, or missing/incomplete data. Table 1 (right) summarizes the exclusion criteria.

The dataset included demographic and clinical parameters such as age, gender, body mass index (BMI), neck circumference, Epworth Sleepiness Scale (ESS) score, Athens Insomnia Scale (AIS) score, smoking status, Fagerström Test for Nicotine Dependence (FTND), and presence of cardiovascular or metabolic comorbidities. Baseline characteristics are presented in Table 2.

The study protocol was approved by the Ethics Committee of the Clinical Hospital of Pulmonary Diseases Iași (approval no. 123, 6 March 2025). Written informed consent was obtained from all participants at the time of their clinical evaluation. This research was conducted as a retrospective observational analysis based on clinical records of patients undergoing diagnostic evaluations between 1 January 2024 and 31 December 2024. All data were collected during routine medical care and stored in the hospital database before the start of the study. No data were accessed, extracted, or analyzed prior to receiving ethical approval; the analysis began only after approval was granted, following institutional and international ethical standards.

The second phase of the study is a prospective follow-up, currently ongoing, designed to monitor changes in inflammatory markers under CPAP therapy at 3, 6, 9, and 12 months after treatment initiation. A key objective of this phase is to evaluate the longitudinal effect of treatment adherence on systemic inflammation, with subgroup comparisons between smokers and non-smokers.

### 1.3. Sleep Test in Our Study

Study participants underwent respiratory polygraphy using the SleepDoc Porti 9 (Löwenstein Medical, Germany), a type III device, performed either during a single night of hospitalization or at home. According to the American Academy of Sleep Medicine (AASM) criteria, obstructive sleep apnea (OSA) was defined as an Apnea–Hypopnea Index (AHI) of ≥5 events per hour. OSA severity was classified as mild (5 ≤ AHI < 15), moderate (15 ≤ AHI < 30), and severe (AHI ≥ 30). Apneas were defined as ≥90% reduction in airflow for at least 10 s. Hypopneas were defined as a 40–50% reduction in respiratory signals for at least 10 s, accompanied by a ≥3% drop in oxygen saturation. All data were manually analyzed and validated by trained sleep physicians. For subgroup analysis, patients were stratified based on AHI, ODI, and mean nocturnal SpO_2_. ODI was categorized as low (<10 events/hour), moderate (10–30 events/h), and high (>30 events/h). Mean nocturnal oxygen saturation was classified as normal (>92%), borderline (88–92%), and hypoxemic (<88%).

### 1.4. Inflammation Assessment

C-reactive protein (CRP) and erythrocyte sedimentation rate (ESR) were included as markers of inflammation. Blood samples were collected from each patient to determine the levels of CRP and ESR. In the Haematology and Biochemistry Laboratory of the Clinical Hospital of Pulmonary Disease in Iasi, standardized procedures were followed for collecting ESR and CRP samples to ensure accuracy and reliability. Samples were collected between 7 and 10 a.m. using sterile, single-use equipment and analyzed within 30 min of collection. For ESR testing, the sample was collected in a tube containing sodium citrate as an anticoagulant. In contrast, CRP testing required serum, which is usually obtained by collecting blood in a clot-activator tube and allowing it to coagulate before centrifugation. All samples were carefully labeled and promptly transported to the respective departments under controlled conditions. A CRP value between 0 and 5 mg/L and an ESR value between 2 and 10 mm/h were considered to be within the normal range.

### 1.5. Statistical Analysis

We conducted a longitudinal analysis using IBM SPSS Statistics for Windows, version 26.0 (IBM Corp., Armonk, NY, USA), on participants who underwent polygraphy during their initial check-up to diagnose obstructive sleep apnea (OSA). Multivariate adjustments were performed for potential confounding variables, such as BMI and smoking status, using multiple linear regression models.

## 2. Results

Table 3 presents the clinical, inflammatory, and sleep-related characteristics of the study population, stratified by OSA severity (mild, moderate, and severe), CRP level categories (<1, 1–3, and >3 mg/dL), and smoking status. As OSA severity increased, a progressive rise was observed in BMI, ODI, and inflammatory markers, such as CRP and ERS. Notably, individuals with severe OSA had the highest BMI (37.47 ± 6.77 kg/m^2^) and ODI (52.88 ± 17.22 events/h) values. CRP levels increased substantially from mild (0.73 ± 0.25 mg/dL) to moderate (7.52 ± 10.34 mg/dL) OSA, remaining elevated in the severe group (6.91 ± 9.13 mg/dL). AHI and ODI values also progressively increased across CRP categories, with the highest values observed in the >3 mg/dL group (AHI: 45.87 ± 19.92/h; ODI: 46.23 ± 20.05/h). Additionally, smokers exhibited markedly elevated CRP (10.32 ± 11.69 mg/dL) and ERS (26.20 ± 19.55 mm/h), compared to non-smokers, along with higher mean AHI and ODI scores, reflecting a greater inflammatory burden and more severe sleep-disordered breathing.

A strong positive correlation between AHI and ODI values was observed (r = 0.996, 95% CI: [0.995,0.998], *p* < 0.001, n = 104). This finding highlights the linear relationship between these two variables and confirms their nearly identical behavior. The regression analysis of AHI versus ODI further supports this nearly perfect linear relationship, ensuring the fidelity and accuracy of the diagnosis. According to existing literature, our study found that C-reactive protein (CRP) levels were associated with both AHI and ODI. CRP levels below 1 mg/dL were linked to moderate OSA (AHI: 30.2/h, ODI: 30.46/h). Levels between 1–3 mg/dL were associated with severe forms of OSA (AHI: 41.38/h, ODI: 41.34/h), while levels above 3 mg/dL were found in individuals with the highest number of respiratory events (AHI: 45.87/h, ODI: 46.23/h). These findings support the study’s hypothesis that systemic inflammation is related to the severity of OSA.

**Figure 1:**
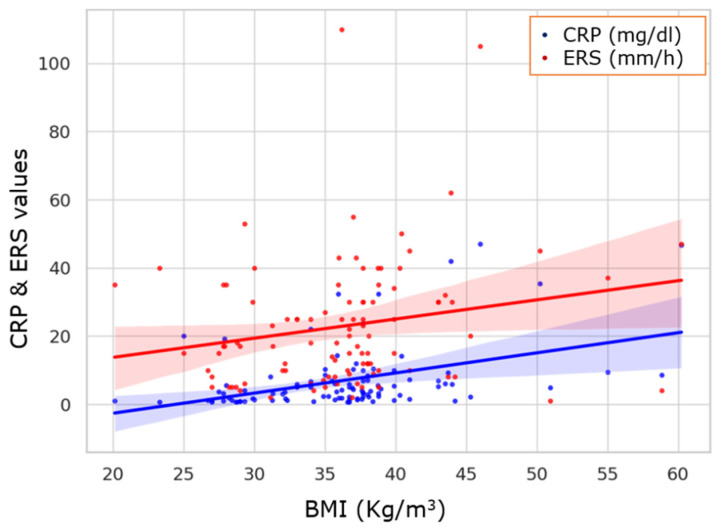
Scatter plot with regression lines for CRP (blue) and ERS (red), compared to BMI. Shaded areas represent 95% confidence intervals. It presents clear upward trends in CRP and ERS values across increasing BMI. It is clear that CRP increases more consistently, while ERS shows greater variability.

Linear regression analysis demonstrated a statistically significant positive association between body mass index (BMI) and CRP levels as shown in Figure 1 (β = 0.585, R^2^ = 0.171, *p* < 0.00). This indicates that approximately 17.1% of the variability in CRP can be explained by changes in BMI, with CRP levels increasing by 0.585 mg/dL for each 1-unit increase in BMI. In a similar manner, a weaker but still statistically positive association was observed between BMI and ERS levels (β = 0.577, R^2^ = 0.043, *p* = 0.035), suggesting that for every 1-unit increase in BMI, ERS may increase by 0.577 mm/h on average. To assess the independent and interactive effects of body mass index (BMI) and smoking on the inflammatory burden in patients with obstructive sleep apnea (OSA), a multivariate linear regression analysis was conducted with CRP as the dependent variable. The model was adjusted for age, sex, diabetes, and cardiovascular disease (CVD) and included an interaction term between BMI and smoking. The model was statistically significant (R^2^ = 0.999, adjusted R^2^ = 0.983, F = 61.46, *p* = 0.016), indicating that a substantial portion of the variance in CRP levels was explained by the included predictors. Both BMI and smoking showed independent associations with higher CRP values, and several BMI and smoking interaction terms were statistically significant, suggesting a possible synergistic inflammatory effect among individuals with high adiposity and active smoking status. A similar multivariate analysis was performed using AHI as the dependent variable. Although the model yielded a high R^2^ = 0.984, the effects of BMI, smoking, and their interaction did not reach statistical significance after adjusting for age, sex, diabetes, and CVD. These findings suggest that while both obesity and smoking are strongly associated with systemic inflammation, their direct influence on OSA severity, as measured by AHI, may be more complex or require larger samples to detect.

**Figure 2:**
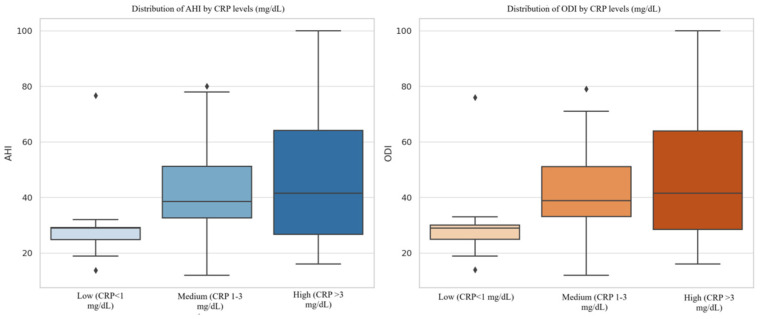
Box plots illustrating the distribution of AHI and ODI values across CRP-level categories. Both plots demonstrated a progressive increase in the median values from low (<1 mg/dL) to high (>3 mg/dL) CRP groups. Moreover, the interquartile range and total spread expanded with higher CRP levels, particularly in the high group, indicating increased heterogeneity in OSA severity. These visual trends align with the statistical findings from ANOVA, reinforcing the link between systemic inflammation and OSA severity.

Variance analysis, performed using a one-way ANOVA test, demonstrated a statistically significant association between CRP levels and the severity of OSA. AHI values differed significantly across CRP groups (F(2, 101) = 4.48, *p* = 0.014), as did ODI values (F(2, 101) = 4.70, *p* = 0.011), like in Figure 2. Specifically, the mean AHI values were 30.20 ± 17.19/h for the <1 mg/dL group, 41.38 ± 20.52/h for the 1–3 mg/dL group, and 45.87 ± 19.92/h for the >3 mg/dL group. Corresponding mean ODI values were 30.46 ± 17.52/h, 41.34 ± 20.06/h, and 46.23 ± 20.05/h, respectively. A progressive increase in OSA severity with higher CRP levels was confirmed by these findings, supporting the claim that systemic inflammation contributes to more severe disease presentations. Post hoc Tukey HSD tests revealed that AHI and ODI values were significantly higher in patients with CRP > 3 mg/dL than those with CRP < 1 mg/dL (*p* = 0.011 and *p* = 0.008, respectively). Differences between the 1–3 mg/dL and >3 mg/dL groups were not statistically significant (*p* > 0.05). This pattern suggests increased heterogeneity in OSA severity among individuals with heightened inflammatory responses.

In order to assess the independent contributions of BMI and smoking, a multivariate linear regression analysis was performed. The model was statistically significant (F(2, 101) = 18.66, *p* < 0.001, R^2^ = 0.270) in determining that BMI (β = 0.491, *p* < 0.001) and smoking (β = 6.002, *p* < 0.001) remained independently associated with CRP levels. The observation confirms that obesity and tobacco consumption contribute significantly to systemic inflammation in OSA-diagnosed subjects.

Higher BMI was associated with increased CRP levels. This was confirmed by the group-wise differences observed in the dataset: individuals with CRP levels < 1 mg/dL had a mean BMI of 31.00 ± 4.75, those with CRP levels between 1–3 mg/dL had a mean BMI of 34.67 ± 5.39, and those with CRP levels > 3 mg/dL had a mean BMI of 38.23 ± 6.84, indicating a progressive increase in BMI across different CRP categories. Regarding ERS levels, although the data exhibited more dispersion, an upward mild trend was noticed. These findings support the idea that adipose tissue influences inflammatory markers.

**Figure 3:**
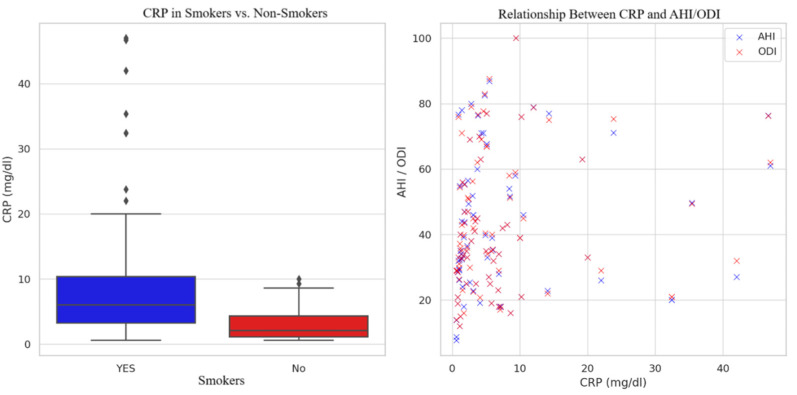
**Left:** Box plot comparing CRP levels in smokers and non-smokers, showing higher median and greater variability in the smoker group. **Right:** Scatter plot illustrating a positive association between CRP levels and AHI/ODI values, indicating that higher systemic inflammation is linked to increased OSA severity. This pattern suggests an association between tobacco use and increased severity and variability in obstructive sleep apnea (OSA) indices.

A comparative statistical analysis was conducted to explore the relationship between tobacco consumption and both the Apnea–Hypopnea Index (AHI) and Oxygen Desaturation Index (ODI). The data revealed that smokers had significantly higher mean values of AHI and ODI, compared to non-smokers (*p* < 0.05). Specifically, smokers exhibited a mean AHI value of 45.29 ± 20.94 events/h and a mean ODI value of 45.69 ± 21.05 events/h, whereas non-smokers recorded lower mean values of 38.40 ± 19.84 events/h for AHI and 38.44 ± 19.40 events/h for ODI. The sample included 55 smokers and 49 non-smokers, ensuring a relatively balanced comparison between groups. Figure 3 depicts these changes.

Furthermore, the analysis demonstrated that smokers had higher levels of CRP (10.32 ± 11.69) compared to non-smokers (2.97 ± 2.45), indicating an elevated inflammatory state among tobacco users with severe forms of OSA. On average, CRP concentrations were approximately 3.5 times higher in smokers. Moreover, smokers also tended to have higher average AHI values (45.29 ± 20.94/h) and ODI values (45.69 ± 21.05/h) than non-smokers (38.40 ± 19.84/h and 38.44 ± 19.40/h, respectively). These findings were strengthened by the fact that the comparison groups were similar in size (55 smokers vs. 49 non-smokers), ensuring comparability.

## 3. Conclusions

This study highlighted a significant association between obstructive sleep apnea (OSA) severity and systemic inflammation, as reflected by elevated C-reactive protein (CRP) and erythrocyte sedimentation rate (ESR) levels. A strong linear relationship between AHI and ODI values was demonstrated, supporting the reliability of both parameters in quantifying the OSA burden.

CRP levels showed a progressive increase alongside worsening OSA severity, confirming the inflammatory component of the disease. In alignment with literature data, which states that excess adipose tissue promotes systemic inflammation, BMI positively correlated with CRP and ERS. Moreover, tobacco use was independently associated with significantly higher AHI, ODI, and CRP values, suggesting that smoking exacerbates the inflammatory states and the severity of OSA. On average, CRP concentrations were approximately 3.5 times higher in smokers.

Both BMI and smoking are independent predictors of elevated CRP levels in OSA patients; thus, they can be considered a contributing factor in desire progression, as the multivariate regression analysis confirmed. The provided data support the hypothesis that both obesity and smoking intensify the inflammatory response in OSA, having the potential to increase cardiovascular and metabolic complications.

Our findings are in line with recent large-scale studies, including a 2023 Mendelian randomization analysis by Liu et al. that employed a Mendelian randomization (MR) approach to investigate the potential causal relationship between smoking and obstructive sleep apnea (OSA). Using summary-level data from extensive genome-wide association studies (GWAS), the researchers selected genetic variants strongly associated with smoking behaviors, including smoking initiation, cigarettes per day, and a lifetime smoking index, as instrumental variables. These were then analyzed against genetic data related to OSA risk derived from a separate GWAS meta-analysis involving individuals of European ancestry. The primary analysis, conducted using inverse variance weighted (IVW) methods, demonstrated that genetically predicted lifetime smoking exposure was significantly associated with a 28% increased risk of developing OSA [21]. A study analyzing data from 3442 participants in the 2020 Korean National Health and Nutrition Examination Survey examined the relationship between smoking and obstructive sleep apnea (OSA). The findings revealed that, among men, both current and former smokers had higher odds ratios for OSA, compared to non-smokers. When data regarding women were evaluated, elevated odds ratios for OSA were also observed, although the associations were more nuanced, particularly about smoking cessation status and pack-years. Among male participants, smoking was significantly associated with a moderate risk of OSA in current smokers and a severe risk in former smokers [22]. Zeng et al. conducted a comprehensive meta-analysis including over 13,000 participants, of whom 3654 were smokers, to investigate the relationship between smoking and obstructive sleep apnea (OSA). The findings showed that smokers had significantly higher Apnea–Hypopnea Index (AHI) values, compared to non-smokers. Additionally, the smoking group demonstrated higher scores on the Epworth Sleepiness Scale (ESS) and lower minimum oxygen saturation (SaO_2_) levels, suggesting more severe OSA and increased daytime sleepiness [23]. These data are in alignment with our findings, thus suggesting that smoking may enhance the inflammatory status associated with OSA.

Clinicians should consider routinely assessing CRP levels in OSA patients, especially smokers or obese ones, to evaluate the inflammatory burden better and guide personalized treatment strategies.

### 3.1. Study Limitations

Naturally, the present study had significant limitations. As noted in the literature, the tendency of the inflammatory syndrome in patients with OSA was to regress within a specific time frame from the initiation of positive pressure therapy. This interval was dictated by the severity class of the disease and associated factors, such as obesity or smoking. In our study, only a percentage of the initially evaluated patients benefited from dynamic biological assessments. We are talking about 25 patients who had tests collected at the 3- or 6-month follow-up. Although statistically irrelevant, we can still observe a trend toward reducing inflammatory markers in patients who underwent biological reassessment.

Furthermore, according to the existing data, this study also observed a negative impact of smoking. Specifically, in our cohort, non-smoking patients were associated with a correction of inflammation markers, in contradiction with the smokers. Similarly, the adverse effect of obesity was noted from this perspective.

Another study limitation was the absence of pack-year data because the binary classification of smoking status (smoker versus non-smoker) was not able to determine the dose-dependent effects of tobacco on the analyzed markers and OSA severity, due to the lack of consistency recovering accurate data from the medical records. The self-reported smoking status may introduce recall bias or misclassification, especially in former or occasional smokers. This aspect was determined by the fact that tailed quantifications of the pack-years could not consistently be retrieved from the available patient records.

High-sensitivity CRP may be more relevant in detecting subclinical chronic low-grade systemic inflammation, as it can detect a minimal increase in CRP levels. As a result, the analysis might miss subtle but meaningful differences between smokers versus non-smokers. In light of these factors, subjects with mild OSA or non-smokers may also have high levels of inflammatory markers that were not detected using standard CRP measurement. Despite these limitations, we consider the data presented as supporting the link between smoking, inflammation, and OSA severity.

Another limitation of this study was the lack of assessment of a broader spectrum of inflammatory markers. Recent evidence suggests that markers such as interleukin-6 (IL-6) and pentraxin-3 (PTX-3) are elevated in patients with obstructive sleep apnea (OSA), even in the absence of hypertension. Furthermore, patients with both OSA and hypertension exhibit an exaggerated inflammatory response and more severe endothelial dysfunction, compared to those with only OSA. As our study did not measure IL-6 and PTX-3, directly assess endothelial function, or stratify patients based on hypertension status, we could not explore these potentially important differential inflammation patterns. Future research should aim to include a broader panel of inflammatory biomarkers and consider comorbid cardiovascular conditions to better characterize the inflammatory landscape of OSA [24].

### 3.2. Further Directions

This study remains open, and in the future, an increasing number of included patients will benefit from the re-evaluation of inflammatory markers at specific periods of time (3, 6, 9 and 12 months). The 25% of patients who underwent evaluation after CPAP initiation belong to the prospective arm of the study, which aims to assess the impact of CPAP use on inflammatory status, as measured by ESR and CRP. This second part of the study has been ongoing since 6 March 2025.

As we know, the gold standard treatment for moderate to severe OSA consists of continuous positive airway pressure (CPAP) devices to maintain the patency of the upper airways. On a secondary level, considering the patients’ profile, the cause of the disease onset, the degree of obesity, and the ENT involvement, there are also other therapeutic approaches. These include prosthetic devices, mandibular advancement strategies, ENT surgery, and surgical obesity treatment. Sleep apnea is associated with “low-grade systemic inflammation”, and therapy may influence its status. Regarding the inflammatory status and the treatment impact, a recent study showed that, compared to healthy controls, individuals with moderate to severe OSA were found to have elevated baseline levels of inflammatory markers, as evaluated via CRP (more than 3.4 mg/L). After the positive pressure titration, CPAP therapy was initiated for one month. At the follow-up visit, patients who exhibited adherence (using therapy for more than four hours on at least 70% of nights) underwent a new evaluation of their inflammatory level. In the non-CPAP group, the inflammatory status worsened, compared to patients who received therapy. However, after one month of treatment, no statistically significant difference was found, as inflammatory levels remained similar, compared with the baseline assessment. Thus, CPAP therapy may have a favorable impact by preventing a further increase in inflammatory markers over time [25]. The usage of CPAP therapy for six months as a treatment for severe OSA, in the case of 37 adherent individuals, revealed a decrease in levels of inflammatory markers, especially in the case of the neutrophil–lymphocyte ratio, fibrinogen-to-albumin ratio, CRP-levels, and fibrinogen, which experienced a statistically significant reduction, thus reinforcing the benefits of CPAP use. Some modifications to CRP levels were evaluated in this paper. A decrease was shown but was not statistically significant [26]. About the long-term use of CPAP devices, Shamed et al. evaluated their impact on high-sensitivity CRP (hs-CRP) after six months of therapy. A decrease in serum hs-CRP levels was observed in individuals with moderate to severe OSA who underwent CPAP therapy for six months (from 4.11 to 2.90 mg/L). The same decrease was maintained among subjects with normal blood pressure (from 3.57 to 2.60 mg/L), whereas, in hypertensive individuals, hs-CRP levels dropped, but not to a statistically significant extent. Conversely, subjects who did not receive CPAP therapy experienced a slight increase in inflammatory marker levels. As stated before, an alternative treatment for OSA involves using oral appliances, such as mandibular advancement devices, with or without mandibular protrusion. Their effect on inflammatory markers, including high-sensitivity C-reactive protein (hs-CRP), interleukin-6, interleukin-10, and TNF-α, was recorded in different papers. In a recent study, after three months of using the device in hypertensive subjects, despite a significant improvement in the Apnea–Hypopnea Index (AHI), there were no significant changes in the levels of circulating inflammatory markers [27]. Kundel et al. conducted a study in which individuals diagnosed with moderate to severe OSA were divided into three inflammatory groups: low, middle, and high. CPAP therapy was then administered for 3 to 4 months. Interestingly, the overall levels of inflammatory markers did not exhibit significant changes. However, there was a 16% increase in inflammatory marker levels in the low-inflammatory group, in contrast to a 20% decrease in the high-inflammatory cluster. It was concluded that CPAP therapy may be effective in reducing cardiovascular disease risk in patients with a high baseline inflammatory phenotype [28]. Elevated blood pressure is commonly observed because the inflammatory cascade associated with sleep apnea impacts the vascular system. In a study involving 86 hypertensive subjects, a comparison of CPAP therapy versus inactive CPAP over 18 weeks revealed significant improvements in nighttime blood pressure, as well as in 24 h ambulatory monitoring. Notably, in addition to blood pressure improvements, CPAP treatment also resulted in a significant reduction in inflammatory markers, including CRP, after 18 weeks [29]. The 14-day CPAP withdrawal was correlated with the return of apneic events, while cardiovascular dysfunction markers, such as endocan, endothelin-1, resistin, and vascular endothelial growth factor, remained at baseline levels. Adrenomedullin, a vasoactive peptide that regulates the cardiovascular system, fluid balance, and immune response, experienced a significant reduction in its levels [30]. The literature data presented above suggest a decline in inflammatory status in a time-dependent manner. Future investigations with expanded patient cohorts and longitudinal biological reassessments will be crucial to solidify these observations and refine our understanding of the interplay between smoking, obesity, and inflammation in the context of sleep apnea. This research is highly relevant to the scientific process, as it provides a foundation for hypothesis-driven inquiries into personalized treatment strategies and the long-term management of sleep apnea. By reinforcing the detrimental role of smoking on inflammatory pathways, it strengthens the existing literature, offering a clearer framework for clinicians and researchers to address modifiable risk factors in this patient population.

In summary, the literature data presented above serve as a starting point for the next phase of the study. We aim to prospectively explore the effects of CPAP on the evolution of inflammatory markers through serial biological reassessments at 3, 6, 9, and 12 months to clarify the dynamic relationship between CPAP adherence and systemic inflammation and to better define the therapeutic potential in mitigating cardiovascular risk in patients with OSA.

## Figures and Tables

**Table 1:** Inclusion criteria (left) and exclusion criteria (right).

Inclusion Criteria	Exclusion Criteria
Age ≥ 18 years	Chronic pulmonary disease (COPD, asthma)
CRP and ESR levels available at the time of diagnosis	Active infections (respiratory, urinary, or other)
AHI ≥ 5 events/hour	Known autoimmune or inflammatory disease affecting CRP/ESR
Documented smoking history	Prior OSA diagnosis or CPAP treatment
	Incomplete data (missing CRP or ESR values, smoking history)

**Table 2:** Baseline characteristics of the study population (n = 104). The mean age was 58.3 years, with a mean BMI of 36.3 kg/m, indicating a predominantly obese population. The average AHI and ODI values were both around 42 events per hour. CRP (6.86 ± 9.38 mg/dL) and ERS (22.62 ± 18.45 mm/h) were elevated. Roughly half of the participants were smokers (50.9%), and common comorbidities included cardiovascular disease and diabetes mellitus, which are consistent with the metabolic risk profile often observed in OSA patients. The mean Epworth Sleepiness Scale (ESS) score was 11.1 ± 5.3, indicating that excessive daytime sleepiness was common among participants, consistent with the symptomatic burden of moderate to severe OSA. The Athens Insomnia Scale (AIS) score averaged 4.32 ± 1.66, suggesting that a notable proportion of participants experienced insomnia symptoms that often coexist with sleep-disordered breathing. Among smokers, the Fagerström Test for Nicotine Dependence (FTND) yielded a high mean score of 7.8 ± 1.3, reflecting substantial nicotine addiction. These questionnaire results highlighted the combined burden of sleepiness, insomnia, and tobacco dependence in this cohort, underlining the multidimensional impact of OSA.

Variable	n	Mean ± SD	Median	Range
Age (years)	104	58.27 ± 13.36	60.5 (52.0–68.0)	4–80
BMI (kg/m^2^)	104	36.33 ± 6.63	36.7 (32.2–38.8)	20.1–60.2
Neck Circumference (cm)	104	41.74 ± 4.20	42.0 (40.1–43.2)	14.0–53.1
Systolic BP (mmHg)	104	72.92 ± 8.15	74.0 (67.0–80.0)	51–90
Diastolic BP (mmHg)	104	129.98 ± 14.42	128.0 (120.0–142.3)	107–160
AHI (events/h)	104	42.05 ± 20.62	37.3 (26.1–55.6)	7.7–100.0
ODI (events/h)	104	42.28 ± 20.52	37.6 (28.2–56.1)	8.3–100.0
CRP (mg/dL)	104	6.86 ± 9.38	3.75 (1.58–7.13)	0.6–47.0
ERS (mm/h)	104	22.62 ± 18.45	18.0 (9.0–30.5)	1–110
ESS Score	104	11.10 ± 5.31	11.0 (6.75–14.0)	3–24
AIS Score	104	4.32 ± 1.66	4.0 (3.0–5.0)	1–8
FTND (Smokers only)	55	7.80 ± 1.32	8.0 (7.0–9.0)	4–10
Nocturnal O_2_ Saturation (%)	104	3.49 ± 15.09	0.92 (0.90–0.94)	0.63–94.2

**Table 3:** The table summarizes the distribution of BMI, CRP, ERS, AHI and ODI values across OSA severity levels, CRP categories and smoking status.

		BMI (Mean ± SD)	CRP (Mean ± SD) (mg/dL)	ERS (Mean ± SD) (mm/h)	AHI (Mean ± SD) (Events/h)	ODI (Mean ± SD) (Events/h)
OSA Severity	Mild OSA (5 ≤ AHI < 15)	26.95 ± 2.57	0.73 ± 0.25	17.00 ± 16.51	10.75 ± 2.73	
	Moderate OSA (15 ≤ AHI < 30)	35.09 ± 5.56	7.52 ± 10.34	23.16 ± 17.22	23.68 ± 5.01	
	Severe OSA (AHI ≥ 30)	37.47 ± 6.77	6.91 ± 9.13	22.70 ± 19.29	52.88 ± 17.22	
CRP Group	CRP < 1 mg/dL				30.20 ± 17.19	30.46 ± 17.52
	CRP 1–3 mg/dL				41.38 ± 20.52	41.34 ± 20.06
	CRP > 3 mg/dL				45.87 ± 19.92	46.23 ± 20.05
Smoking Status	Smokers		10.32 ± 11.69	26.20 ± 19.55	45.29 ± 20.94	45.69 ± 21.05
	Non-Smokers		2.97 ± 2.45	18.66 ± 16.41	38.40 ± 19.84	38.44 ± 19.40

## Data Availability

Data is contained within the article.

## References

[1] Kim J., In K., Kim J., You S., Kang K., Shim J., Lee S., Lee J., Lee S., Park C. (2004). Prevalence of sleep-disordered breathing in middle-aged Korean men and women. Am. J. Respir. Crit. Care Med..

[2] Senaratna C.V., Perret J.L., Lodge C.J., Lowe A.J., Campbell B.E., Matheson M.C., Hamilton G.S., Dharmage S.C. (2017). Prevalence of obstructive sleep apnea in the general population: A systematic review. Sleep Med. Rev..

[3] Benjafield A.V., Ayas N.T., Eastwood P.R., Heinzer R., Ip M.S.M., Morell M.J., Nunez C.M., Patel S.R., Penzel T., Pepin J.-L. (2019). Estimation of the global prevalence and burden of obstructive sleep apnea: A literature-based analysis. Lancet Respir. Med..

[4] Gölen M.K., Isik S.M., Arikan V. (2024). Is there a relationship between the severity of obstructive sleep apnea syndrome and the systemic immune-inflammation index?. Eur. Arch. Otorhinolaryngol..

[5] Labarca G., Gower J., Lampeti L., Dreyse J., Jorquera J. (2020). Chronic intermittent hypoxia in obstructive sleep apnea: A narrative review from pathophysiological pathways to a precision clinical approach. Sleep Breath..

[6] Nadeem R., Molnar J., Madbouly E.M., Nida M., Aggarwal S., Sajid H., Naseem J., Loomba R. (2013). Serum inflammatory markers in obstructive sleep apnea: A meta-analysis. J. Clin. Sleep Med..

[7] Lee W.H., Wee J.H., Rhee C.S., Yoon I.-Y., Kim J.-W. (2016). Erythrocyte sedimentation rate may help predict severity of obstructive sleep apnea. Sleep Breath..

[8] Sanchez-Azofra A., Gu W., Masso-Silva J.A., Sanz-Rubio D., Marin-Oto M., Cubero P., Gil A.V., Moya E.A., Barnes L.A., Mesarwi O.A. (2023). Inflammation biomarkers in OSA, chronic obstructive pulmonary disease, and chronic obstructive pulmonary disease/OSA overlap syndrome. J. Clin. Sleep Med..

[9] Al-Mughales J., Wali S.O., Manzar D., Alhejaili F., Gozal D. (2022). Pro-inflammatory markers in patients with obstructive sleep apnea and the effect of continuous positive airway pressure therapy. Sleep Sci..

[10] Shiels M.S., Katki H.A., Freedman N.D., Purdue M.P., Wentzensen N., Trabert B., Kitahara C.M., Furr M., Li Y., Kemp T.J. (2014). Cigarette smoking and variations in systemic immune and inflammation markers. J. Natl. Cancer Inst..

[11] Lavie L., Lavie P. (2008). Smoking interacts with sleep apnea to increase cardiovascular risk. Sleep Med..

[12] Ioannidou D., Kalamaras G., Kotoulas S.-C., Pataka A. (2021). Smoking and obstructive sleep apnea: Is there an association between these cardiometabolic risk factors?—Gender analysis. Medicina.

[13] Vgontzas A.N., Papanicolaou D.A., Bixler E.O., Kales A., Tyson K., Chrousos G.P. (1997). Elevation of plasma cytokines in disorders of excessive daytime sleepiness: Role of sleep disturbance and obesity. J. Clin. Endocrinol. Metab..

[14] Ciftci T.U., Kokturk O., Bukan N., Bilgihan A. (2004). The relationship between serum cytokine levels with obesity and obstructive sleep apnea syndrome. Cytokine.

[15] Phillips B.G., Kato M., Narkiewicz K., Choe I., Somers V.K. (2000). Increases in leptin levels, sympathetic drive, and weight gain in obstructive sleep apnea. Am. J. Physiol. Heart Circ. Physiol..

[16] Zhang X.L., Yin K.S., Wang H., Xi L., Zhang K., Yin S., Su S. (2006). Plasma adiponectin levels in obstructive sleep apnea–hypopnea syndrome. Chest.

[17] Pataka A., Kotoulas S., Kalamaras G., Tzinas A., Grigoriou I., Kasnaki N., Argyropolou P. (2022). Does smoking affect OSA? What about smoking cessation?. J. Clin. Med..

[18] Kim K.S., Kim J.H., Park S.Y., Won H.R., Lee H.J., Yang H.S., Kim H.J. (2012). Smoking induces oropharyngeal narrowing and increases the severity of obstructive sleep apnea syndrome. J. Clin. Sleep Med..

[19] Conway S.G., Roizenblatt S.S., Palombini L., Castro L.S., Bittencourt L.R.A., Silva R.S., Tufik S. (2008). Effect of smoking habits on sleep. Braz. J. Med. Biol. Res..

[20] Lacedonia D., Salerno F.G., Carpagnano G.E., Sabato R., Depalo A., Foschino-Barbaro M.P. (2011). Effect of CPAP-therapy on bronchial and nasal inflammation in patients affected by obstructive sleep apnea syndrome. Rhinology.

[21] Liu K., Li K., Zang C., Wang J., Liu J., Chen Z., He M., Liu B., Su X., Zhang Y. (2023). Smoking and obstructive sleep apnea: A Mendelian randomization study. Sleep Med. X..

[22] Jang Y.S., Nerobkova N., Hurh K., Park E.-C., Shin J. (2023). Association between smoking and obstructive sleep apnea based on the STOP-Bang index: A nationwide cross-sectional study. Sci. Rep..

[23] Zeng X., Ren Y., Wu K., Yang Q., Zhang S., Wang D., Luo Y., Zhang N. (2022). Association between smoking behavior and obstructive sleep apnea: A systematic review and meta-analysis. Nicotine Tob. Res..

[24] Damiani M.F., Zito A., Carratu P., Falcone V.A., Bega E., Scicchitano P., Ciccone M.M., Resta O. (2015). Obstructive sleep apnea, hypertension, and their additive effects on atherosclerosis. Biochem. Res. Int..

[25] Wang X., Guan L., Wu C., Zhao Y., Zhao G. (2023). Continuous positive airway pressure may improve hypertension in patients with obstructive sleep apnea–hypopnea syndrome by inhibiting inflammation and oxidative stress. Arch. Med. Sci..

[26] Turnbull C.D., Rossi V.A., Santer P., Schwarz E.I., Stradling J.R., Petousi N., Kohler M. (2017). Effect of OSA on hypoxic and inflammatory markers during CPAP withdrawal: Further evidence from three randomized control trials. Respirology.

[27] Msaad S., Chaabouni A., Marrakchi R., Boudaya M., Kotti A., Feki W., Jamoussi K., Kammoun S. (2020). Nocturnal continuous positive airway pressure (nCPAP) decreases high-sensitivity C-reactive protein (hs-CRP) in obstructive sleep apnea–hypopnea syndrome. Sleep Disord..

[28] Friščić T., Perčić M., Vidović D., Štajduhar A., Galić E. (2022). Impact of CPAP therapy on new inflammation biomarkers. J. Clin. Med..

[29] Hedberg P., Nohlert E., Tegelberg Å. (2021). Effect of oral appliance treatment on inflammatory biomarkers in obstructive sleep apnea: A randomized controlled trial. J. Sleep Res..

[30] Kundel V., Cohen O., Khan S., Patel M., Kim-Schulze S., Kovacic J., Suares-Farinas M., Shah N.A. (2023). Advanced proteomics and cluster analysis for identifying novel obstructive sleep apnea subtypes before and after continuous positive airway pressure therapy. Ann. Am. Thorac. Soc..

